# Bacteroidetes and Firmicutes Drive Differing Microbial Diversity and Community Composition Among Micro-Environments in the Bovine Rumen

**DOI:** 10.3389/fvets.2022.897996

**Published:** 2022-05-19

**Authors:** Lee J. Pinnell, Arquimides A. Reyes, Cory A. Wolfe, Maggie D. Weinroth, Jessica L. Metcalf, Robert J. Delmore, Keith E. Belk, Paul S. Morley, Terry E. Engle

**Affiliations:** ^1^Veterinary Education, Research, and Outreach Program, Texas A&M University, Canyon, TX, United States; ^2^Department of Animal Sciences, Colorado State University, Fort Collins, CO, United States

**Keywords:** 16S rRNA gene sequencing, cattle, rumen, microbiome, core microbial community

## Abstract

Ruminants are a critical human food source and have been implicated as a potentially important source of global methane emissions. Because of their unique digestive physiology, ruminants rely upon a symbiotic relationship with the complex and rich community of microorganism in the foregut to allow digestion of complex carbohydrates. This study used 16S rRNA gene sequencing to investigate the composition of microbial communities from three rumen micro-environments of cattle fed identical diets: (1) free fluid, (2) the fibrous pack, and (3) the mucosa. Community composition analysis revealed that while a phylogenetic core including the most abundant and most common ruminal taxa (members of Bacteroidetes and Firmicutes) existed across micro-environments, the abundances of these taxa differed significantly between fluid- and mucosa-associated communities, and specific lineages were discriminant of individual micro-environments. Members of Firmicutes, specifically Clostridiales, Lachnospiraceae, Mogibacteriaceae, Christenellaceae, and Erysipelotrichaceae were significantly more abundant in fluid communities, while members of Bacteroidetes, namely Muribaculaceae and Prevotellaceae were more abundant in mucosa-associated communities. Additionally, Methanobacteriaceae, a family of methanogenic Archaea, was more abundant in fluid-associated communities. A set of four more diverse lineages were discriminant of pack-associated communities that included Succinivibrionaceae, RFP12 (Verruco-5), Fibrobacteraceae, and Spirochaetaceae. Our findings indicate that different ecological niches within each micro-environment have resulted in significant differences in the diversity and community structure of microbial communities from rumen fluid, pack, and mucosa without the influence of diet that will help contextualize the influence of other environmental factors.

## Introduction

Ruminant livestock are a critical source of food for humans worldwide, with the population of cattle alone estimated to be over 1.5 billion (1). Ruminants are also of environmental significance as they release considerable amounts of methane into the atmosphere (2). Characterized by their unique method of plant digestion, ruminants rely on a complex consortium of microorganisms to partially digest plant polysaccharides in their forestomach, or rumen before it enters the glandular “true” stomach. Thus, the microbial community within the rumen plays an essential role in the health of the animal by providing a major source of nutrients (3), and the microbiota of the rumen are critical to the productivity of this important food source and to potential significance of methane emissions.

The rumen is a rich and diverse microbial ecosystem largely composed of anaerobic bacteria, protozoa, anaerobic fungi, methanogenic archaea, and phages. Bacteria represent the most abundant and diverse taxonomic group, and it is the bacterial members of the rumen microbial community that primarily drive the degradation and fermentation of plant fibers and proteins into digestible compounds such as volatile fatty acids and microbial proteins (4, 5). Ruminal archaea are mostly limited to methanogenic members of the phylum Euryarchaeota, and more specifically the classes Methanobacteria, Methanomicrobia, and Thermoplasmata. Typically, ruminal microbial communities are predominately made up of starch and sugar degrading organisms (5), though it is well-established that diet exerts a strong influence on ruminal microbial community composition (6–8). Despite this, abundant ruminal taxa are remarkably consistent across individual animals regardless of their diet or location. In a meta-analysis characterizing ruminal microbial communities in 742 individual ruminants from across the world, the top 30 most abundant microbial genera were found in over 90% of samples (9).

The microbiota of the rumen as a whole has been well-characterized, but relatively few studies have investigated differences in community structure between ruminal components. While there are a large number of ecological niches available, broadly speaking rumen microorganisms can be free-living or particle-attached in ruminal fluid, attached to the fibrous pack, or attached to the ruminal mucosa (10). Differences in diversity and community structure have been identified between liquid and solid-associated ruminal communities (5, 11, 12), but comparisons to mucosa- and pack-associated microbial communities are limited (13, 14). Mucosa-associated microbial communities in the rumen have also been described temporally (15) and been compared to ruminal pack-associated communities (16), but there is little knowledge about which microbial taxa are discriminant of ruminal fluid, pack, and mucosa-associated communities.

This study utilized 16S rRNA gene sequencing to characterize the diversity and composition of microbial communities of different locations within the rumen: within the ruminal fluid, fibrous pack, and those found on the mucosa. It was designed to test if the previously described ruminal phylogenetic core is present across ruminal micro-environments, and to discover which microbial taxa were discriminant of these micro-environments. We hypothesized that core rumen taxa would be present across all three micro-environments, but that the composition of fluid, pack, and mucosa-associated microbial communities would differ.

## Materials and Methods

### Cattle Population

Twelve crossbred feedlot steers (450 kg; ~3.0 years of age) fitted with ruminal fistulas, were utilized in this study. All cattle in this study were adjusted to a high energy finishing diet consisting of ~90% concentrate and 10% roughage (1.43 NEg MCal/kg—Supplementary Table S1) for 4 weeks (28 d) before sample collection. Animals were managed and sampled in accordance with Colorado State University's (CSU) Institutional Animal Care and Use Committee (IACUC) approval (Protocol 16-6550A). Steers were housed at CSU's Agricultural Research, Development and Education Center.

### Rumen Content Collection

At the completion of the diet adjustment period, samples from three micro-environments within the rumen were collected *via* ruminal fistulas from each steer ~1 h after morning feeding: the aqueous portion of the rumen (fluid; *n* = 12), a bolus of the ruminal fibrous pack (pack; *n* = 12), and a swab of the ruminal mucosa (mucosa; *n* =12). The bolus of fibrous material was sampled by collecting 85 g from the center of the solid portion of the rumen, and the ruminal mucosa was sampled by running a sterile swab across the ruminal wall. Fluid was collected by compressing the fibrous bolus and capturing the resulting liquid. After collection, samples were immediately placed on ice, transported to the laboratory and stored at −80°C at the laboratory until DNA isolation.

### DNA Isolation, 16S rRNA Library Preparation and Sequencing

Genomic DNA was isolated from fluid and mucosal swab samples using a QIAamp PowerFecal DNA Kit (Qiagen, Hilden, Germany) from 0.16 to 0.18 g of material, while a DNeasy PowerMax Soil Kit was used to isolate DNA from 2 to 5 g fibrous pack samples. Following isolation, DNA was quantified (ng μL^−1^) and assayed for quality (A_260_/A_280_) using a NanoDrop spectrophotometer (Thermo Fisher Scientific, Inc., Waltham, MA).

Amplicon library preparation and sequencing was carried out by Novogene Corporation Inc (Chula Vista, CA, USA). The V4 region of the 16S rRNA gene was amplified with the 515f (5′ – GTG CCA GCM GCC GCG GTA A – 3′) and 806r (5′ – GCA CTA CHV GGG TWT CTA AT – 3′) primer pair and Novogene's proprietary amplification conditions, which include no template PCR negative controls. Following successful PCR, amplicon libraries were prepared and pooled using Novogene's proprietary process and sequenced on an Illumina HiSeq 2500 instrument (Illumina Inc., San Diego, CA, USA) using 2 × 250 bp paired-end chemistry. The number of sequenced reads ranged from 188,276 to 219,029, with an average of 209,279 reads per sample. The average sequencing depth did not differ between the three micro-environments (Pairwise Wilcoxon rank-sum + Benjamini-Hochberg correction for multiple comparisons, *p* > 0.05, *n* = 12).

### Bioinformatics

Demultiplexed paired-end reads were imported into QIIME2 version 2020.11 (16) and DADA2 was used to filter reads for quality, remove chimeric sequences, merge overlapping paired-end reads, and generate amplicon sequence variants (ASVs) (17). Forward reads were trimmed at 17 bp and truncated at 249 bp, while reverse reads were trimmed at 21 bp and truncated at 249 bp. Taxonomy was assigned using a Naïve Bayes classifier trained on the Greengenes version 13_8 99% OTUs database (18), where sequences had been trimmed to include only the base pairs from the V4 region bound by the 515f/806r primer pair. Reads that mapped to chloroplast and mitochondrial sequences were filtered from the representative sequences and ASV table using the “filter_taxa” function, and a midpoint-rooted phylogenetic tree was then generated using the “q2-phylogeny” pipeline with default settings, which was used to calculate phylogeny-based diversity metrics. Data and metadata were then imported into phyloseq (19) using the “import_biom” and “import_qiime_sample_data” functions and merged into a phyloseq object. Richness (Observed ASVs), Shannon's diversity, and Faith's Phylogenetic Diversity (FPD) were calculated for all samples with phyloseq and the “estimate_pd” function from the btools package. Samples were then proportionally transformed to the lowest total ASV count of 101,175 and beta-diversity was analyzed using weighted, generalized, and unweighted UniFrac distances (20, 21). From these distances, non-metric multidimensional scaling (NMDS) was performed and plotted, and a permutational multivariate analysis of variance (PERMANOVA) was used to test for significant differences in community structure using the vegan (22) and pairwiseAdonis (23) packages. To ensure significant differences were not the result of unequal dispersion of variability between groups, permutational analyses of dispersion (PERMDISP) were conducted for all significant PERMANOVA outcomes using the vegan package. Hierarchical clustering was performed using Ward's agglomeration clustering method (24) on generalized UniFrac distances and the “hclust” function. Further, the relative abundances of ASVs within each sample were calculated and plotted using phyloseq. The proportion of reads mapping to each taxonomic rank are displayed in Supplementary Table S1.

### Rumen Phylogenetic Core and Discriminant Lineages

The core microbiota of the rumen was identified at the taxonomic rank of genus. A detection limit (minimum allowed relative abundance at the level of genus) of 0.1% and a minimum prevalence (proportion of samples the genus was present in) of 80% were used as cutoffs, and the “core” function within the package microbiome was used to calculate and plot a heatmap of core community members.

To identify taxa discriminant of each micro-environment (i.e., fluid, pack, mucosa), linear discriminant analysis effect size (LEfSe) was performed using the LEfSe tool (25) with default settings, except for using a more stringent alpha value (0.01) and a higher logarithmic LDA score threshold (4.0) due to small sample size. Because of the low proportion of reads classified at the rank of genus (40.4%; Supplementary Table S1) families with a mean relative abundance >0.5% across all samples were considered, with the factor “micro-environment” set as class, and an “one-against-all” strategy was applied. The relative abundances of the families considered discriminant of any of the three micro-environments were then individually visualized and compared.

### Statistical Analyses

Unless specified otherwise, R version 3.6.3 (26) was used for statistical analysis of data. Pairwise Wilcoxon rank-sum tests were performed with a Benjamini-Hochberg correction (PW+BH) for multiple comparisons. Differences in beta diversity were tested using pairwise PERMANOVA with a Benjamini-Hochberg correction for multiple comparisons and 9,999 permutations. Additionally, pairwise PERMDISPs were carried out for all significant PERMANOVA outcomes using 9,999 permutations to test for differences in the variability of dispersions. Except for the LEfSe analysis, an alpha of 0.05 was used as the cutoff to reject the null hypothesis and for differences to be considered statistically significant.

### Data Availability

All sequence reads were made available through the BioProject PRJNA749669 at the National Center for Biotechnology Information's Sequence Read Archive.

## Results

### Microbial Richness and Diversity

**Figure 1:**
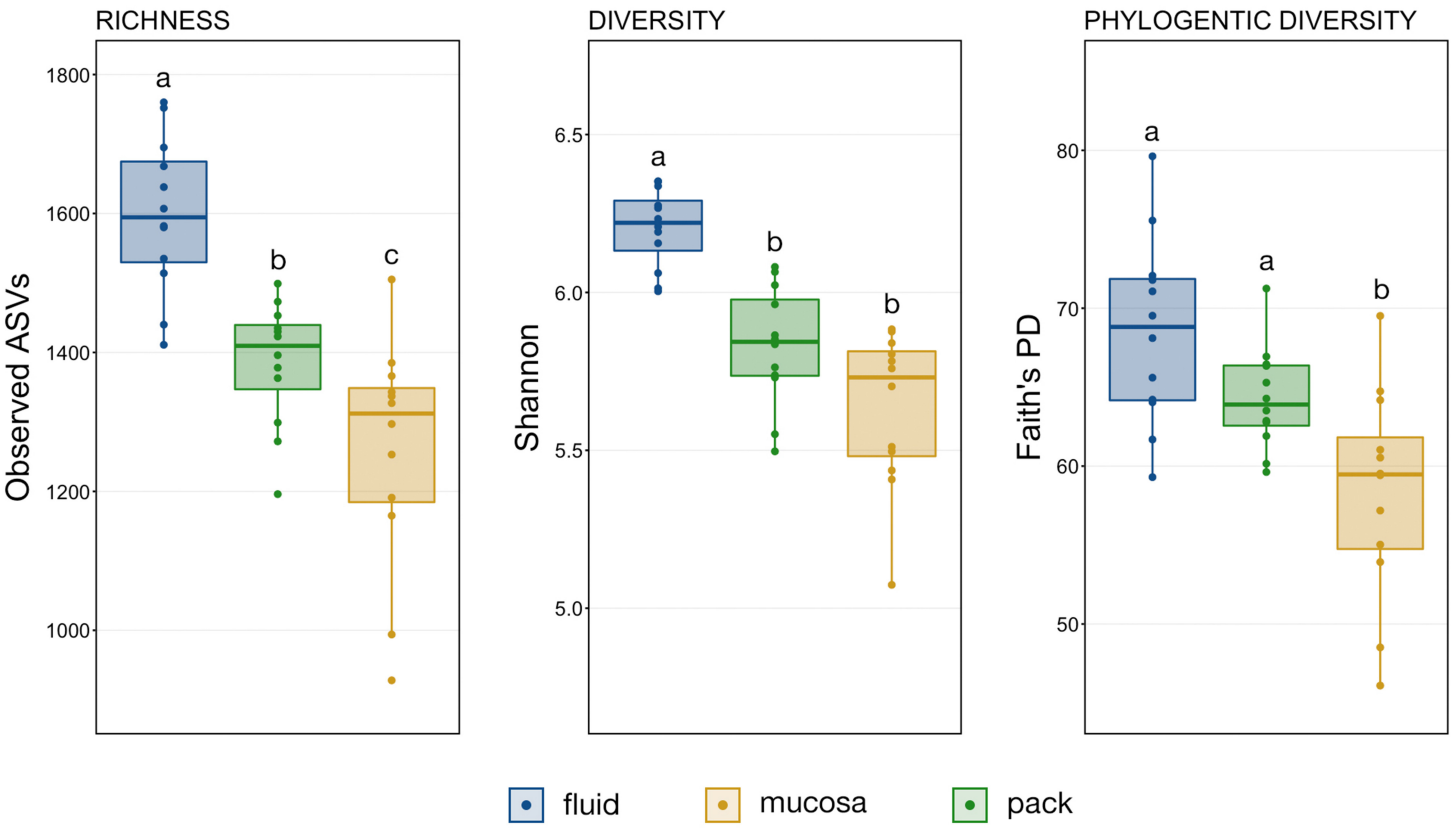
Boxplots displaying the number of observed ASVs and Faith's Phylogenetic Distance for each ruminal component (fluid, pack, mucosa). Significant differences in richness and diversity between components are illustrated by different letters (Pairwise Wilcoxon rank-sum with Benjamini-Hochberg correction, *p* < 0.05, *n* = 12).

A comparison of observed ASVs was used to test for differences in richness among the ruminal components, and comparisons of Shannon's index and Faith's Phylogenetic Distance (FPD) were used to test for differences in diversity. The trend was the same for richness, diversity, and phylogenetic diversity, with fluid being the richest and the most diverse followed by pack and then mucosa (Figure 1). However, statistical significance differed between metrics. Microbial communities within the rumen fluid were richer than ruminal pack and mucosa communities and pack communities were richer than mucosa (Figure 1; PW+BH, *n* = 12, *p* < 0.05). Fluid communities were also more diverse than pack and mucosa communities, which did not differ significantly from each other (Figure 1; PW+BH, *n* = 12, *p* < 0.05). However, the phylogenetic diversity of ruminal fluid and pack communities were not different, and both were more phylogenetically diverse than mucosal communities (Figure 1; PW+BH, *n* = 12, *p* < 0.05).

### Rumen Phylogenetic Core

**Figure 2:**
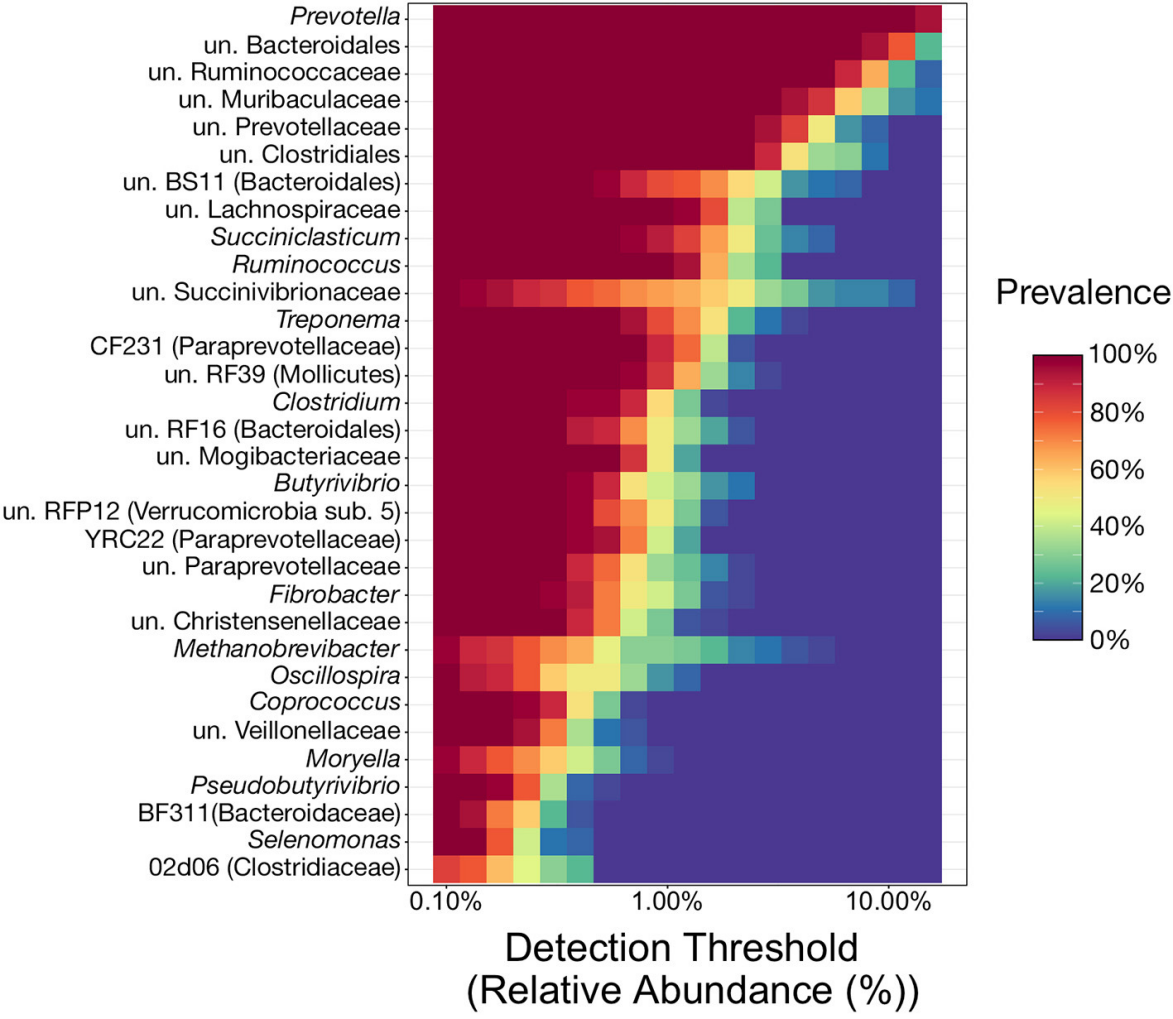
Heatmap illustrating the microbial phylogenetic core of the bovine rumen in feedlot cattle at the taxonomic rank of genus. To be considered core taxa, genera needed to comprise more than 0.1% of the overall microbial community and be present in at least 80% of all samples (*n* = 36). With the relative abundance values on the x-axis, the heatmap demonstrates the prevalence of the 32 genera considered core taxa at differing relative abundance levels (from the 0.1% minimum cutoff to the maximum observed value of 30.6%). This results in genera with high prevalence values at high relative abundances near the top of the heatmap, and those meeting the prevalence cutoff (80%) at lower relative abundances at the bottom. un, unclassified.

Of the 117 genera above the detection threshold of 0.1% relative abundance, 32 (27%) were present in at least 80% of samples from all three ruminal components across every individual animal, and thus were considered to represent the phylogenetic core (Figure 2). Of the genera within the phylogenetic core, 22 (69%) of them belong to the phyla Bacteroidetes or Firmicutes, and specifically the orders Bacteroidales or Clostridiales. Genera from these two orders were the most abundant members of the phylogenetic core, with 9 of the top 10 most abundant core genera belonging to these two orders (Figure 2). Interestingly, of the 32 core genera, all 32 were present in 100% of all samples.

### Microbial Community Structure of Ruminal Components

**Figure 3:**
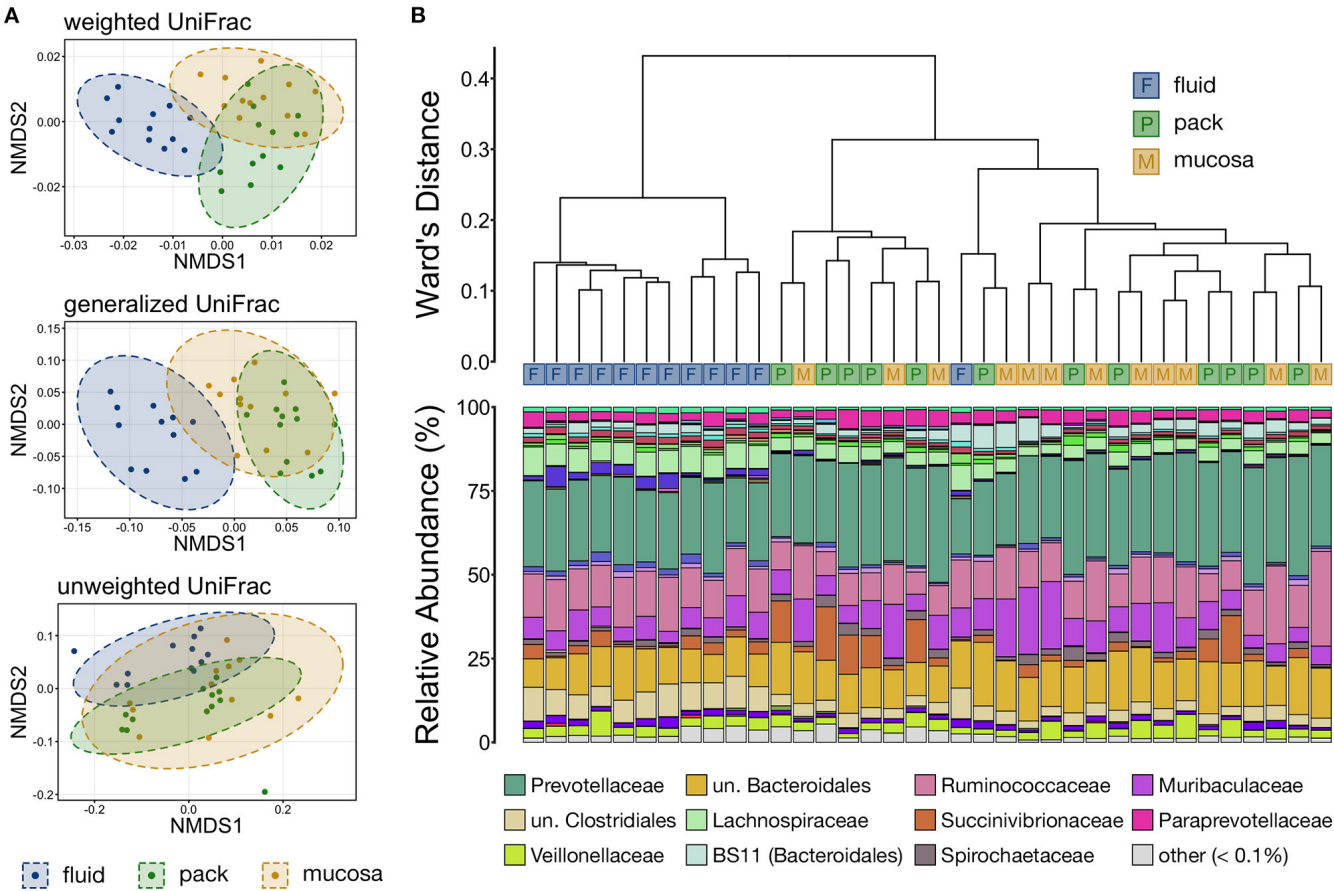
**(A)** Non-metric multidimensional scaling (NMDS) of weighted, generalized, and unweighted UniFrac distances illustrating variation in microbial community structure associated with each ruminal component. The NMDS demonstrates clustering of 16S rRNA gene sequences from rumen fluid, pack, and mucosal microbial communities. Dashed lines and shaded areas represent 95% confidence ellipses for each ruminal micro-environment. **(B)** The relatedness of rumen fluid, pack, and mucosa microbial communities based on normalized ASVs. Hierarchical clustering was performed on generalized UniFrac distances using Ward's agglomeration method. Blue boxes (F) represent fluid communities, green boxes (P) represent pack communities, and gold boxes (S) represent mucosal communities. The barplot illustrates the relative abundance of microbial families within each individual sample. The 12 most abundant families across all samples are displayed in the legend.

Differences in overall microbial community structure among the three ruminal components was analyzed using NMDS, hierarchical clustering, and PERMANOVA. The composition of ruminal fluid, pack, and mucosa communities were different from each other when compared using all three UniFrac distances (Supplementary Table S1; pairwise PERMANOVA, *n* = 12, *p* < 0.05). Non-significant PERMDISP tests confirmed that significant PERMANOVA results were the result of differences in community structure and not due to unequal dispersions of variance (Supplementary Table S1; pairwise PERMDISP, *p* > 0.05). Visualization with NMDS illustrated that microbial communities from each of the three ruminal components were distinct, and that communities became more similar when compared using less weight on abundant lineages (i.e., from weighted to unweighted UniFrac). Further, fluid and pack communities appear to be more tightly clustered and distinct from each other, while mucosa communities were slightly more varied (Figure 3A).

Hierarchical clustering based on generalized UniFrac values revealed that fluid communities were most similar to other fluid communities and formed a fluid-specific clade, with the exception of one fluid community that formed a clade with pack and mucosa communities collected from the same individual animal (Figure 3B). Ruminal pack and mucosa communities were more intermixed, however both pack and mucosa communities were slightly more likely to cluster with other communities from the same ruminal component (i.e., pack or mucosa). The dichotomy between rumen fluid and pack or mucosa communities was primarily the result in differences in the abundances of members of Bacteroidales (higher in pack/mucosa-associated communities) and Clostridiales (higher in fluid-associated communities) (Figure 3B).

### Ruminal Component-Specific Discriminant Lineages

**Figure 4:**
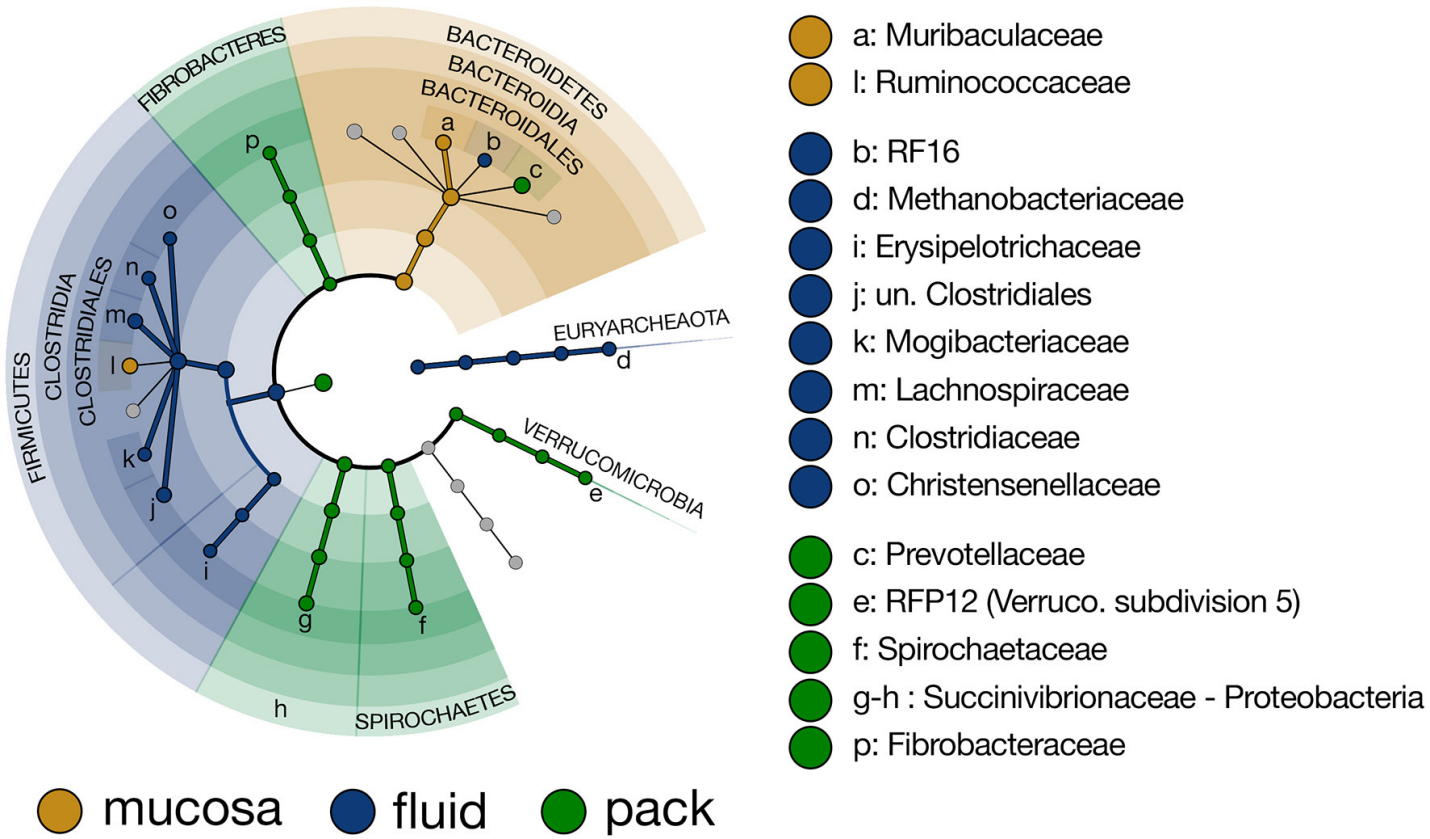
Cladogram demonstrating the microbial families with relative abundances of >0.5% of the total community across fluid, pack, and mucosa-associated microbial communities. Taxa discriminant of fluid communities are highlighted in blue, taxa discriminant of pack communities are highlighted in green, and taxa discriminant of mucosa communities are highlighted in gold (LEfSe, *p* < 0.01, *n* = 12).

To more closely investigate taxa that were responsible for the differing community structure among the three ruminal components, linear discriminant analysis effect size (LEfSe) was used to identify taxonomic lineages that were differentially abundant between rumen fluid, pack, and mucosa communities. Results show that six lineages of Firmicutes (unclassified Clostridiales, Lachnospiraceae, Clostridiaceae, Mogibacteriaceae, Christensenellaceae, and Erysipelotrcihaceae) were discriminant of fluid communities. Five of the six lineages included members of Clostridiales. Further, Methanobacteriaceae (Euryarchaeota) and RF16 (Bacteroidales) were discriminant of fluid-associated communities (Figure 4). Muribaculaceae (Bacteroidales) and Ruminococcaceae (Clostridiales) were discriminant of mucosa-associated communities (Figure 4), though Ruminococcaceae being discriminant of mucosal communities is misleading. Ruminococcaceae was in similar abundance in fluid and mucosa-associated communities (see **Figure 8** below), and its designation as mucosa-discriminant was an artifact of the “one-against-all” comparison strategy coupled with a lower abundance in pack communities. A set of four more diverse lineages including RFP12 (Verrucomicrobia), Spirochaetaceae (Spirochaetes), Succinivibrionaceae (Proteobacteria), and Fibrobacteraceae (Fibrobacteres) were discriminant of pack communities (Figure 4).

**Figure 5:**
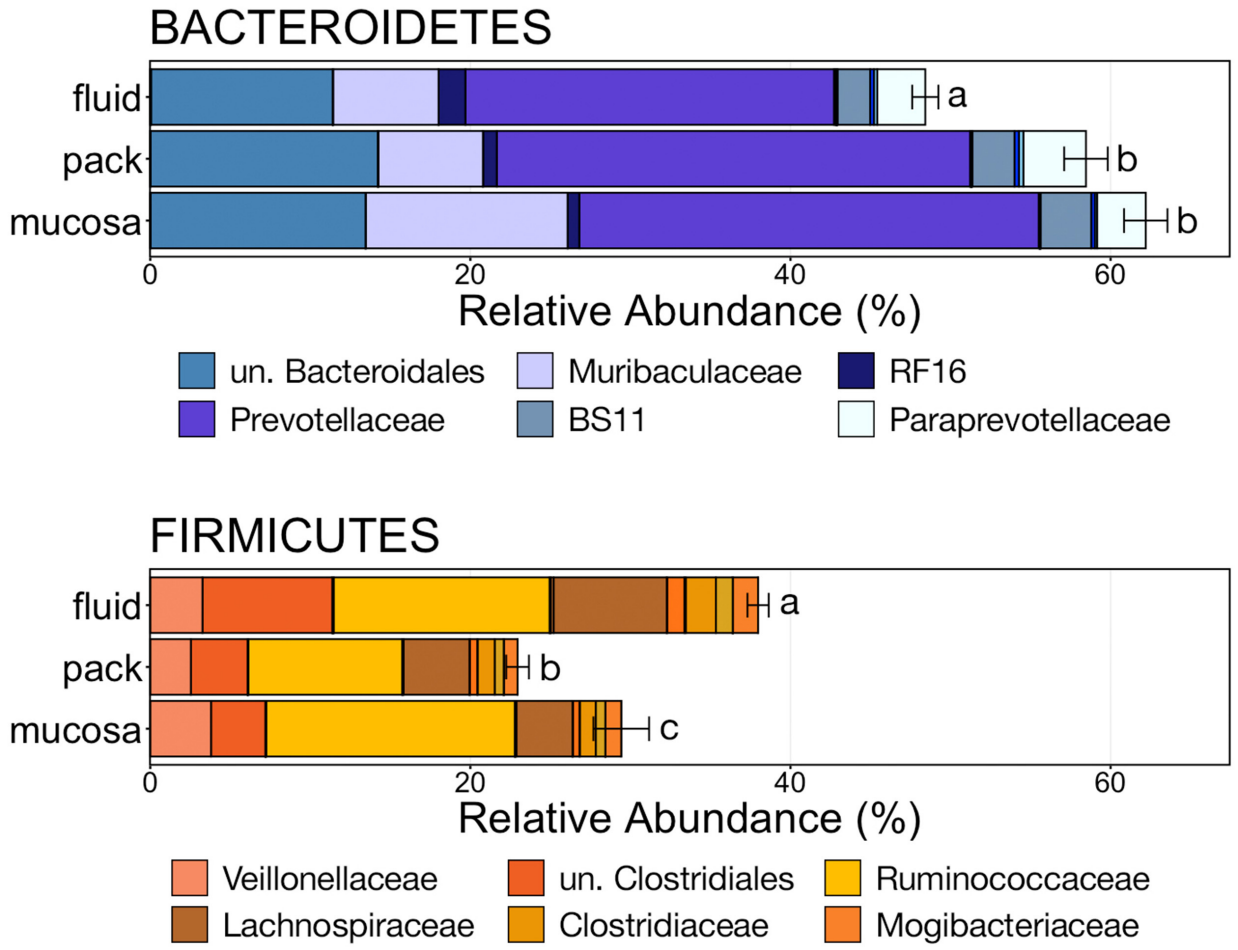
Bar plot showing the relative abundances of the phyla Bacteroidetes and Firmicutes. Error bars display the standard error of the mean for each phylum and colors represent the relative abundance of families of the two phyla within rumen fluid, pack, and mucosa-associated communities. The top six most abundant families within each phylum are displayed in the legend. Significant differences in the relative abundance of the two phyla are illustrated by different letters (Pairwise Wilcoxon rank-sum with Benjamini-Hochberg correction, *p* < 0.05, *n* = 12). un, unclassified.

**Figure 6:**
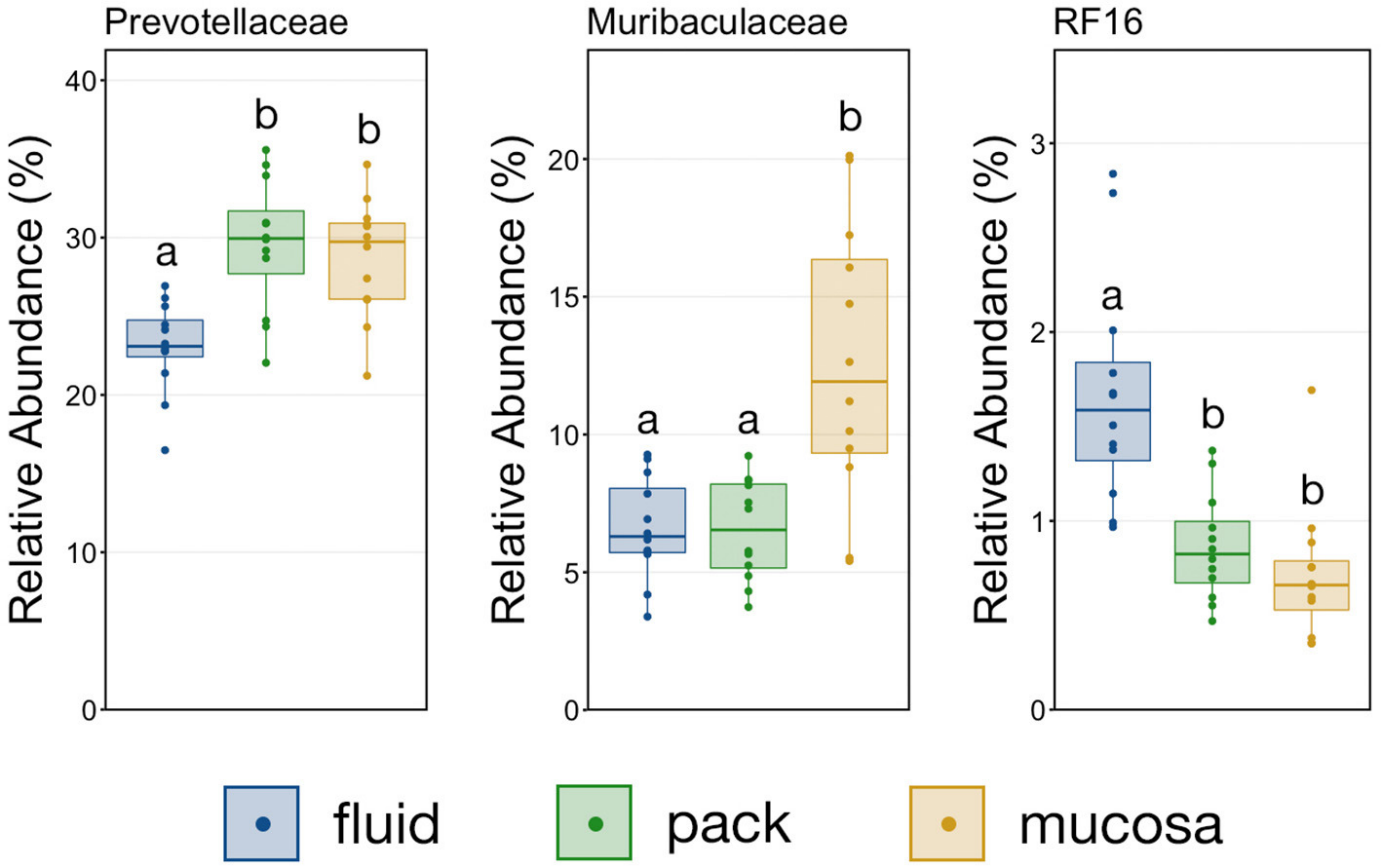
Boxplot demonstrating the relative abundances of the three families from the phylum Bacteroidetes discriminant of a ruminal micro-environments. Points represent individual animals, and the horizontal line in each boxplot represents the median relative abundance. Significant differences between the relative abundances of each family are illustrated by different letters (Pairwise Wilcoxon rank-sum with Benjamini-Hochberg correction, *p* < 0.05, *n* = 12).

To further characterize the Bacteroidetes-Firmicutes dichotomy among fluid, pack, and mucosa communities, the relative abundances of each phylum and the families comprising them were directly compared. The abundance of members of Bacteroidetes was higher in mucosa- and pack-associated communities than fluid communities (Figure 5; PW+BH, *n* = 12, *p* < 0.05). Differences in Bacteroidetes were largely the result of differences within the families of Prevotellaceae and Muribaculaceae (Figure 5), which represented the two most abundant families of Bacteroidetes. Prevotellaceae was less abundant in fluid communities compared to both pack and mucosa, while Muribaculaceae was more abundant in mucosa-associated communities vs. both fluid and pack (Figure 6; PW+BH, *n* = 12, *p* < 0.05). RF16 (Bacteroidales) was more abundant in fluid communities (Figure 6; PW+BH, *n* = 12, *p* < 0.05), although its relative abundance was far lower than that of Prevotellaceae and Muribaculaceae in all three micro-environments.

**Figure 7:**
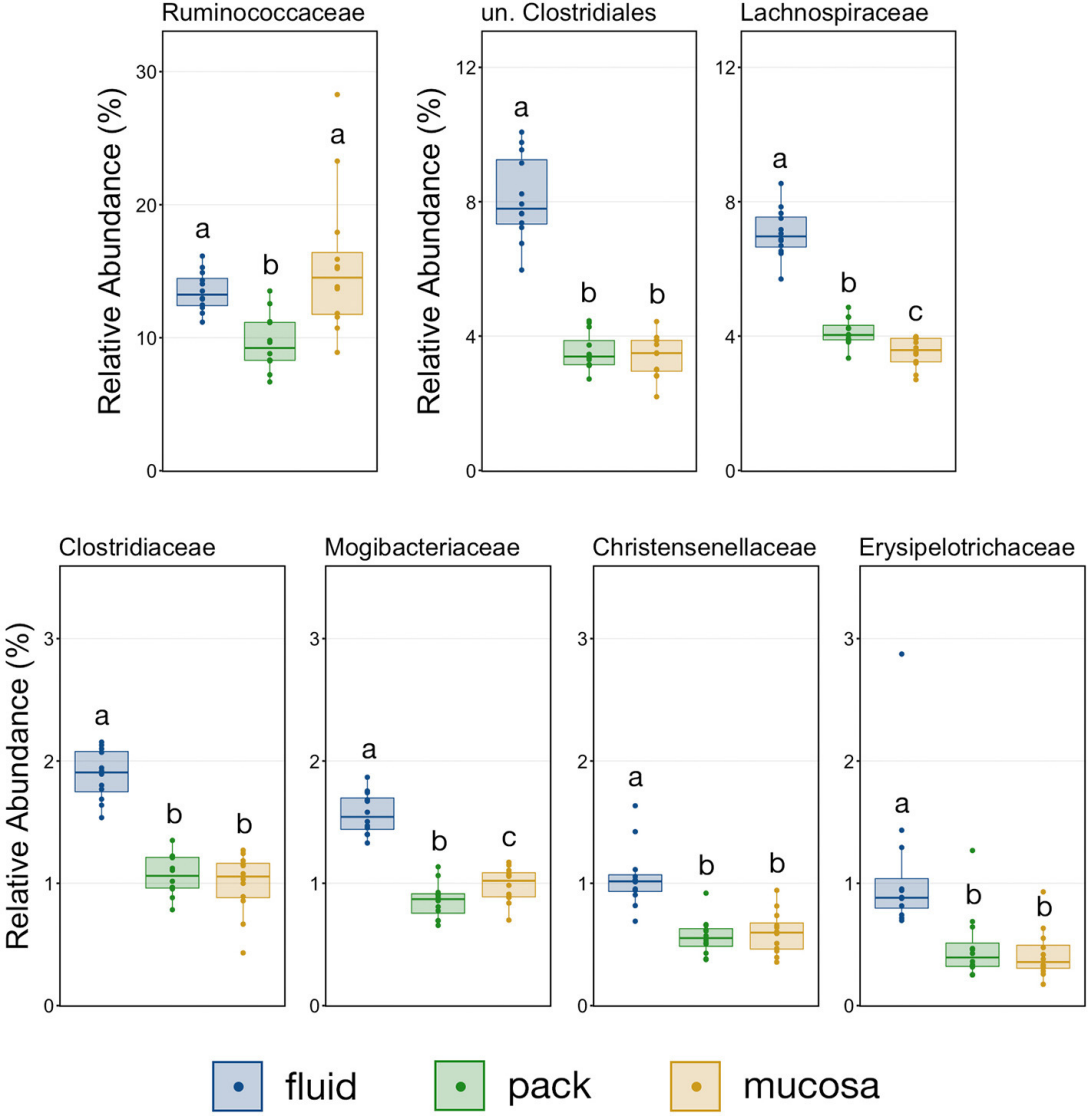
Boxplot demonstrating the relative abundances of the seven families from the phylum Firmicutes discriminant of a ruminal micro-environments. Points represent individual animals, and the horizontal line in each boxplot represents the median relative abundance. Significant differences between the relative abundances of each family are illustrated by different letters (Pairwise Wilcoxon rank-sum with Benjamini-Hochberg correction, *p* < 0.05, *n* = 12).

The relative abundance of Firmicutes was different among each of the three ruminal components, with the phylum being more abundant in fluid communities than both pack and mucosa-associated communities, and less abundant in mucosa-associated communities then pack (Figure 5; PW+BH, *n* = 12, *p* < 0.05). The differential abundance of Firmicutes between ruminal components was largely the result of differences in the relative abundance unclassified Clostridiales and Lachnospiraceae (Figure 5). A total of six families within Firmicutes (unclassified Clostridiales, Lachnospiraceae, Clostridiaceae, Mogibacteriaceae, Christensenellaceae, Erysipelotrichaceae) were more abundant in fluid communities then both pack and mucosa-associated communities, while Ruminococcaceae was more abundant in fluid and mucosa-associated communities then in pack (Figure 7; PW+BH, *n* = 12, *p* < 0.05).

**Figure 8:**
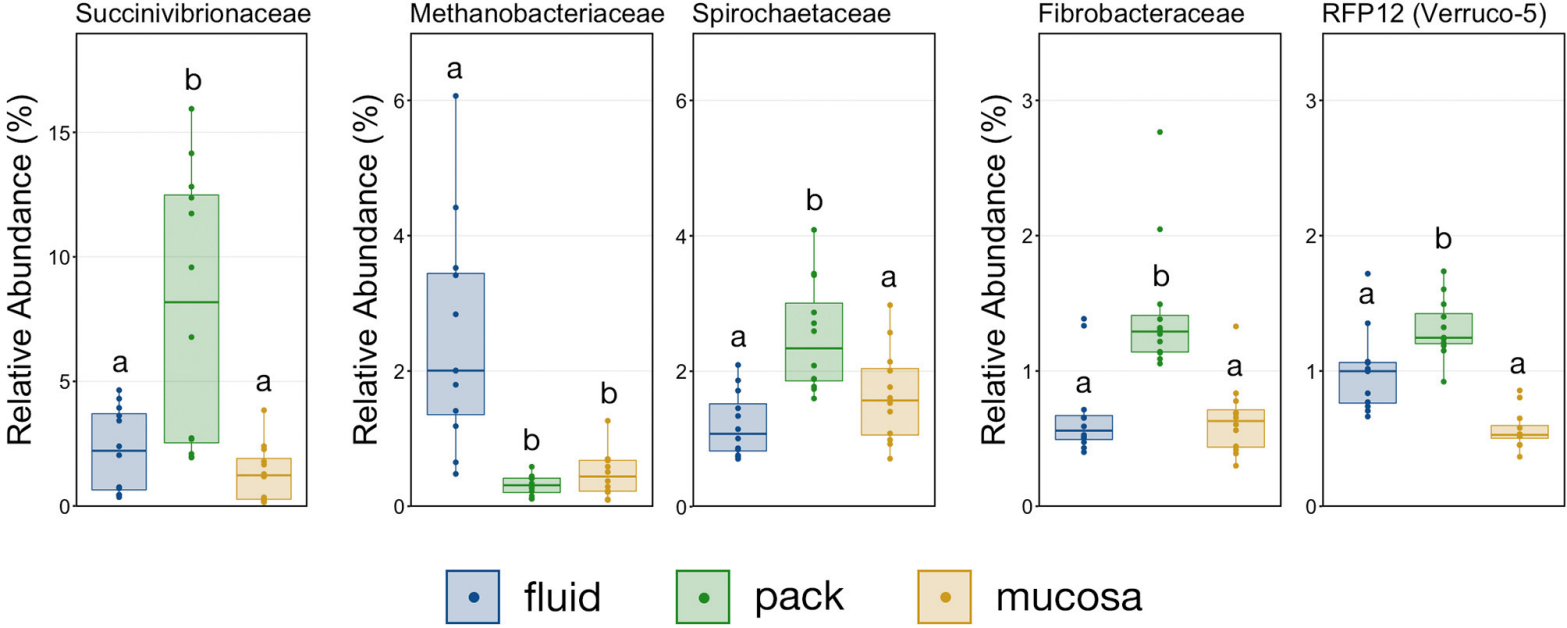
Boxplot demonstrating the relative abundances of the five families outside of Bacteroidetes and Firmicutes that are discriminant of a ruminal micro-environments. Points represent individual animals, and the horizontal line in each boxplot represents the median relative abundance. Significant differences between the relative abundances of each family are illustrated by different letters (Pairwise Wilcoxon rank-sum with Benjamini-Hochberg correction, *p* < 0.05, *n* = 12).

With the exception the Methanobacteriaceae, which was discriminant of fluid-associated communities (Figure 4), every discriminant lineage outside of Firmicutes and Bacteroidetes was from pack-associated communities. Succinivibrionaceae, RFP12, Spirochaetaceae, and Fibrobacteraceae were all discriminant of pack-associated communities and belong to the phyla Proteobacteria, Verrucomicrobia, Spirochaetes, and Fibrobacteres, respectively (Figure 5). Pack-associated communities had higher relative abundances of Succinivibrionaceae, Spirochaetaceae (virtually entirely made up of *Treponema*), Fibrobacteraceae, and RFP12 then fluid and mucosa-associated communities, which had similar abundances (Figure 8; PW+BH, *n* = 12, *p* < 0.05). The family Methanobacteriaceae was in higher abundance in fluid-associated communities than both pack and mucosa-associated communities (Figure 8; PW+BH, *n* = 12, *p* < 0.05).

## Discussion

This study used 16S rRNA gene sequencing to investigate the localization of microbiota within the bovine rumen and to identify microbial taxa discriminant of its micro-environments. Results demonstrated that rumen fluid contained the richest and most diverse microbial communities, while mucosa-associated communities were the least diverse. Importantly, while a large proportion of microbial taxa were present in every ruminal fluid, pack, and mucosa-associated community, the proportions of these taxa differed significantly among ruminal components. Members of the phylum Firmicutes were disproportionately predominant within fluid communities as compared to pack and mucosa-associated communities, while members of the phylum Bacteroidetes were disproportionately under-represented in fluid communities. Further, pack-associated communities contained discriminant lineages from the phyla Proteobacteria, Verrucomicrobia, Spirochaetes, and Fibrobacteres. Diet-induced shifts within rumen microbial communities are well-established (5–8) and due to the consistent diet throughout this study, these results provide a baseline for the diversity and community composition of microbial communities associated with specific ruminal micro-environments (i.e., fluid, pack, and mucosa) that can be used to contextualize the influence of environmental factors such as diet.

Rumen fluid functions as a transportation and growth medium for digestive microorganisms and as a result it could be expected that that majority of microbial taxa found within the rumen as a whole would be present in the fluid even if in very low abundance. Indeed, fluid communities in this study were the richest, but diversity was similar in fluid and pack-associated communities. Higher richness, but similar diversity suggests that ruminal fluid contains more types of microorganisms, but the additional types are either rare or closely related. Interestingly, mucosa-associated communities in this study were both less rich and less diverse, which contradicts previous research that found mucosal communities to be most diverse (13). However, multiple differences in methodology (i.e., different primers, lower sequencing depth, rarefied data, OTU/ASV picking method) could explain the differences. For example, the previous study had considerably lower sequence depth [490,001 processed sequences across 89 samples (5,505 sequences per sample)] as compared to this study [4,571,185 processed sequences across 36 samples (126,977 sequences per sample)]. Further, the previous study performed OTU clustering with an unusually low similarity cutoff (95%), while this study denoised sequences to produce ASVs without the need to cluster, which provides a higher resolution examination of the community structure (17). Differences in community coverage (i.e., sequencing depth) and sequence processing could explain the contradictory relationship between micro-environments.

A large number of studies have used amplicon sequencing to characterize the rumen's phylogenetic core and illustrated the overwhelming dominance of the phyla Bacteroidetes and Firmicutes, and more specifically members of orders Bacteroidales and Clostridiales (9, 27–29). Here, we demonstrated that these two orders remain very abundant and were found in 100% of samples collected from ruminal fluid, pack, and mucosa-associated communities. Our finding that 27% of taxa were included in the phylogenetic core across the three micro-environments falls within the range of previous research investigating the ruminal phylogenetic core in whole-rumen samples (29–31). The shared core between each micro-environment is in itself largely uninteresting, as it only illustrates the presence of microbial taxa and not abundance.

Despite the most abundant taxa being dominant in all three micro-environments, fluid, pack, and mucosa-associated microbial communities were different when compared with and without weight being placed on abundance, suggesting that both abundant and rare taxa contribute to the differences. Variations in community composition in communities associated with liquid- and solid-fractions of different ruminal micro-environments have been well-described (5, 12, 13) and this study confirms this. Further, our results illustrate that the difference between liquid- and solid-associated fractions is much larger than that between solid- and mucosa-associated communities. The dichotomy between free-living and attached microbial communities is a long-established fact in environmental microbial ecology (32–34) and here we demonstrate this likely holds true in the rumen as well.

The primary drivers of the liquid-solid dichotomy in the rumen were members of the phyla Bacteroidetes and Firmicutes. In particular, members of Muribaculaceae (formerly S24-7) and Prevotellaceae (both families of Bacteroidetes) were more abundant in solid attached communities, with Muribaculaceae abundance being the highest in mucosal communities. A study of the Muribaculaceae family showed that its members have high functional diversity with regards to complex carbohydrate degradation, and contain protective mechanisms against benzoate and macrolide antibiotics (35). However, the family is relatively poorly characterized and why it is over-represented in the mucosa specifically should be the focus of future investigation. Contrastingly, a set of six families within Firmicutes were significantly more abundant in fluid-associated communities. The most abundant of these belonged to the Clostridiales (unclassified Clostridiales, Lachnospiraceae, and Clostridiaceae). Lachnospiraceae is a well-characterized butyrate producer (36), which is an important energy source for rumen epithelial cells and plays a role in regulating rumen barrier function (37). Lachnospiraceae produce butyrate through the degradation of plant fibers (38), and would benefit from access to the pack. Additionally, ruminal fluid itself contains small fiber particles and would provide an environment where Lachnospiraceae and other butyrate producers would be in contact with both a fiber-source and the epithelium where the butyrate would be used as an energy source. Comparisons of the abundance of Bacteroidetes and Firmicutes between liquid and solid rumen communities have produced mixed results; either reporting very small differences between fluid and solids (5), or more drastic differences similar to ours (13).

It should also be noted that the samples were collected from cattle that had permanent fistulas placed through the skin into the rumen. Accessing the rumen *via* these fistulas meant that mucosal samples were taken from the surface by swab, and it was not possible to determine whether the mucosa was bathed with ruminal fluid during collection, which could represent a source of cross-contamination between micro-environments. However, it is unlikely that these factors would have been fully responsible for creating systematic differences among the microbiota found in the different ruminal fractions that were study. Another consideration is that fluid samples here were collected from fluid extracted from the pack, which could potentially yield different communities than free fluid found in the ventral rumen. One additional potentially confounding factor in this study was the use of different extraction methods for fluid and solid communities (pack and mucosa-associated), which has been demonstrated to influence 16S rRNA amplicon sequencing and subsequent community composition results in ruminal communities (39, 40). While two extraction kits were used, they both used mechanical shearing (bead-beating) without phenol and both kits utilize the same chemistries, which eliminates the largest source of extraction bias (39). Despite this, community structure (i.e., relative abundance of different taxa) may have been influenced by extraction method and resulted in differences in relative abundance values. However, we believe any influence was likely minimal and would not change the conclusions drawn from the results of this study.

Multiple lineages outside Bacteroidetes and Firmicutes were also discriminant of individual micro-environments. With the exception of Methanobacteriaceae, a methanogenic Euryarcheaota, lineages outside the two major phyla were discriminant of pack-associated communities. Members of Spriochaetes are known to be involved with the degradation of soluble fibers (41), and members of Fibrobacteraceae are widely described ruminal flora capable of degrading plant-based cellulose (42, 43). It follows that members of Spirochaetaceae and Fibrobacteraceae would be over-represented in communities attached to the fibrous pack. Members of the family Succinivibrionaceae ferment carbohydrate to produce succinate and acetate (44) and were also was discriminant of pack-associated communities. Interestingly, this family has been linked to lower methane emissions (45). Contrastingly, the family RFP12 from the phylum Verrucomicrobia was discriminant of pack-associated communities but has been negatively correlated with lower methane emissions (46) and Methanobacteriaceae, known methane producers in the rumen (47, 48), were over-represented in fluid-associated communities. Our results suggest that efforts to promote or negate the growth of these families implicated in methane emissions should target the flora that are abundant in the ruminal fibrous pack and fluid.

## Data Availability Statement

The datasets presented in this study can be found in online repositories. The names of the repository/repositories and accession number(s) can be found below: https://www.ncbi.nlm.nih.gov/, BioProject PRJNA749669.

## Ethics Statement

The animal study was reviewed and approved by Colorado State University's (CSU) Institutional Animal Care and Use Committee.

## Author Contributions

KB, PM, TE, JM, RD, MW, and AR participated in and provided oversight of all aspects of the study including securing funding, design, laboratory procedures, data analysis, and report preparation. CW participated in study conduct and laboratory analyses. LP performed the data analysis and wrote the manuscript. All authors read, edited, and approved the final manuscript.

## Funding

This study received funding from MicroBios Inc. The funder was not involved in the study design, collection, analysis, interpretation of data, the writing of this article or the decision to submit it for publication.

## Conflict of Interest

The authors declare that the research was conducted in the absence of any commercial or financial relationships that could be construed as a potential conflict of interest.

## Publisher's Note

All claims expressed in this article are solely those of the authors and do not necessarily represent those of their affiliated organizations, or those of the publisher, the editors and the reviewers. Any product that may be evaluated in this article, or claim that may be made by its manufacturer, is not guaranteed or endorsed by the publisher.

## References

[1] FAO. World Food and Agriculture - Statistical Yearbook 2020. Rome, Italy (2020).

[2] McMichael AJ Powles JW Butler CD Uauy R. Food, livestock production, energy, climate change, and health. Lancet. (2007) 370:1253–63. 10.1016/S0140-6736(07)61256-2 17868818

[3] Kamra DN. Rumen microbial ecosystem. Curr Sci. (2005) 89:124–35. 10.2307/24110438

[4] Brulc JM Antonopoulos DA Miller MEB Wilson MK Yannarell AC Dinsdale EA. Gene-centric metagenomics of the fiber-adherent bovine rumen microbiome reveals forage specific glycoside hydrolases. Proc Natl Acad Sci USA. (2009) 106:1948–53. 10.1073/pnas.0806191105 19181843 PMC2633212

[5] Deusch S Camarinha-Silva A Conrad J Beifuss U Rodehutscord M Seifert J. A Structural and functional elucidation of the rumen microbiome influenced by various diets and microenvironments. Front Microbiol. (2017) 8:1605. 10.3389/fmicb.2017.01605 28883813 PMC5573736

[6] Lengowski MB Witzig M Möhring J Seyfang GM Rodehutscord M. Effects of corn silage and grass silage in ruminant rations on diurnal changes of microbial populations in the rumen of dairy cows. Anaerobe. (2016) 42:6–16. 10.1016/j.anaerobe.2016.07.004 27451293

[7] Pitta DW Pinchak WE Dowd SE Osterstock J Gontcharova V Youn E. Rumen bacterial diversity dynamics associated with changing from bermudagrass hay to grazed winter wheat diets. Microb Ecol. (2010) 59:511–22. 10.1007/s00248-009-9609-6 20037795

[8] Zhang R Zhu W Liu J Mao S. Effect of dietary forage sources on rumen microbiota, rumen fermentation and biogenic amines in dairy cows. J Sci Food Agricult. (2014) 94:1886–95. 10.1002/jsfa.6508 24375419

[9] Henderson G Cox F Ganesh S Jonker A Young W Global Rumen Census C. Rumen microbial community composition varies with diet and host, but a core microbiome is found across a wide geographical range. Sci Rep. (2015) 5:14567. 10.1038/srep14567 26449758 PMC4598811

[10] Hungate RE. The Rumen and Its Microbes. New York, NY: Academic Press (1966).

[11] de Menezes AB Lewis E O'Donovan M O'Neill BF Clipson NE Doyle EM. Microbiome analysis of dairy cows fed pasture or total mixed ration diets. FEMS Microbiol Ecol. (2011) 78:256–65. 10.1111/j.1574-6941.2011.01151.x 21671962

[12] Kong Y Teather R Forster R. Composition, spatial distribution, and diversity of the bacterial communities in the rumen of cows fed different forages. FEMS Microbiol Ecol. (2010) 74:612–22. 10.1111/j.1574-6941.2010.00977.x 21044097

[13] AlZahal O Li F Guan LL Walker ND McBride BW. Factors influencing ruminal bacterial community diversity and composition and microbial fibrolytic enzyme abundance in lactating dairy cows with a focus on the role of active dry yeast. J Dairy Sci. (2017) 100:4377–93. 10.3168/jds.2016-11473 28390722

[14] Stockler RM Hallowell H Higgins KV Groover ES Hiltbold EM Newcomer B. Characterization and comparison of the rumen luminal and epithelial microbiome profiles using metagenomic sequencing technique. Front Vet Sci. (2022) 9:799063. 10.3389/fvets.2022.799063 35280141 PMC8907629

[15] Guo W van Niekerk JK Zhou M Steele MA Guan LL. Longitudinal assessment revealed the shifts in rumen and colon mucosal-attached microbiota of dairy calves during weaning transition. J Dairy Sci. (2021) 104:5948–63. 10.3168/jds.2020-19252 33612210

[16] Bolyen E Rideout JR Dillon MR Bokulich NA Abnet CC Al-Ghalith GA. Reproducible, interactive, scalable and extensible microbiome data science using QIIME 2. Nat Biotechnol. (2019) 37:852–7. 10.1038/s41587-019-0209-9 31341288 PMC7015180

[17] Callahan BJ McMurdie PJ Rosen MJ Han AW Johnson AJ Holmes SP. DADA2: high-resolution sample inference from Illumina amplicon data. Nat Methods. (2016) 13:581–3. 10.1038/nmeth.3869 27214047 PMC4927377

[18] DeSantis TZ Hugenholtz P Larsen N Rojas M Brodie EL Keller K. Greengenes, a chimera-checked 16S rRNA gene database and workbench compatible with ARB. Appl Environ Microbiol. (2006) 72:5069–72. 10.1128/AEM.03006-05 16820507 PMC1489311

[19] McMurdie PJ Holmes S. phyloseq: an R package for reproducible interactive analysis and graphics of microbiome census data. PLoS ONE. (2013) 8:e61217. 10.1371/journal.pone.0061217 23630581 PMC3632530

[20] Chen J Bittinger K Charlson ES Hoffmann C Lewis J Wu GD. Associating microbiome composition with environmental covariates using generalized UniFrac distances. Bioinformatics. (2012) 28:2106–13. 10.1093/bioinformatics/bts342 22711789 PMC3413390

[21] Lozupone C Lladser ME Knights D Stombaugh J Knight R. UniFrac: an effective distance metric for microbial community comparison. ISME J. (2011) 5:169–72. 10.1038/ismej.2010.133 20827291 PMC3105689

[22] Oksanen J Blanchet FG Friendly M Kindt R Legendre P McGlinn D. vegan: Community Ecology Package. R package version 2.5-5. ed. (2019). Available online at: https://CRAN.R-project.org/package=vegan (accessed October 9, 2019).

[23] Arbizu PM. pairwiseAdonis: pairwise multilevel comparison using adonis. (2017). Available online at: https://github.com/pmartinezarbizu/pairwiseAdonis (accessed October 9, 2019).

[24] Murtagh F Legendre P. Ward's hierarchical agglomerative clustering method: which algorithms implement Ward's criterion? J Classif. (2014) 31:274–95. 10.1007/s00357-014-9161-z

[25] Segata N Izard J Waldron L Gevers D Miropolsky L Garrett WS. Metagenomic biomarker discovery and explanation. Genome Biol. (2011) 12:R60. 10.1186/gb-2011-12-6-r60 21702898 PMC3218848

[26] R Core Team. R: A Language and Environment for Statistical Computing. Vienna, Austria: R Foundation for Statistical Computing (2017).

[27] Petri RM Schwaiger T Penner GB Beauchemin KA Forster RJ McKinnon JJ. Characterization of the core rumen microbiome in cattle during transition from forage to concentrate as well as during and after an acidotic challenge. PLoS ONE. (2014) 8:e83424. 10.1371/journal.pone.0083424 24391765 PMC3877040

[28] Wallace RJ Sasson G Garnsworthy PC Tapio I Gregson E Bani P. A heritable subset of the core rumen microbiome dictates dairy cow productivity and emissions. Sci Adv. (2019) 5:eaav8391. 10.1126/sciadv.aav8391 31281883 PMC6609165

[29] Wirth R Kádár G Kakuk B Maróti G Bagi Z Szilágyi Á. The planktonic core microbiome and core functions in the cattle rumen by next generation sequencing. Front Microbiol. (2018) 9:2285. 10.3389/fmicb.2018.02285 30319585 PMC6165872

[30] Jami E Mizrahi I. Composition and similarity of bovine rumen microbiota across individual animals. PLoS ONE. (2012) 7:e33306. 10.1371/journal.pone.0033306 22432013 PMC3303817

[31] Xue M Sun H Wu X Guan LL Liu J Nojiri H. Assessment of rumen microbiota from a large dairy cattle cohort reveals the pan and core bacteriomes contributing to varied phenotypes. Appl Environ Microbiol. (2018) 84:e00970–18. 10.1128/AEM.00970-18 30054362 PMC6146982

[32] Crump BC Armbrust EV Baross JA. Phylogenetic analysis of particle-attached and free-living bacterial communities in the Columbia River, its estuary, and the adjacent coastal ocean. Appl Environ Microbiol. (1999) 65:3192–204. 10.1128/AEM.65.7.3192-3204.1999 10388721 PMC91474

[33] DeLong EF Franks DG Alldredge AL. Phylogenetic diversity of aggregate-attached vs. free-living marine bacterial assemblages. Limnol Oceanogr. (1993) 38:924–34. 10.4319/lo.1993.38.5.0924

[34] Hollibaugh JT Wong PS Murrell MC. Similarity of particle-associated and free-living bacterial communities in northern San Francisco Bay, California. Aquat Microb Ecol. (2000) 21:103–14. 10.3354/ame021103

[35] Lagkouvardos I Lesker TR Hitch TCA Gálvez EJC Smit N Neuhaus K. Sequence and cultivation study of Muribaculaceae reveals novel species, host preference, and functional potential of this yet undescribed family. Microbiome. (2019) 7:28. 10.1186/s40168-019-0637-2 30782206 PMC6381624

[36] Vital M Howe AC Tiedje JM Moran MA. Revealing the bacterial butyrate synthesis pathways by analyzing (meta)genomic data. mBio. (2014) 5:e00889-14. 10.1128/mBio.00889-14 24757212 PMC3994512

[37] Shen H Xu Z Shen Z Lu Z. The regulation of ruminal short-chain fatty acids on the functions of rumen barriers. Front Physiol. (2019) 10:1305. 10.3389/fphys.2019.01305 31749707 PMC6842973

[38] Cotta M Forster R. (2006). The family Lachnospiraceae, including the genera *Butyrivibrio, Lachnospira* and *Roseburia*. In: Dworkin M, Falkow S, Rosenberg E, Schleifer KH, Stackebrandt E, editors. The Prokaryotes. New York, NY: Springer. 10.1007/0-387-30744-3_35

[39] Henderson G Cox F Kittelmann S Miri VH Zethof M Noel SJ. Effect of DNA extraction methods and sampling techniques on the apparent structure of cow and sheep rumen microbial communities. PLoS ONE. (2013) 8:e74787. 10.1371/journal.pone.0074787 24040342 PMC3770609

[40] Villegas-Rivera G Vargas-Cabrera Y González-Silva N Aguilera-García F Gutiérrez-Vázquez E Bravo-Patiño A. Evaluation of DNA extraction methods of rumen microbial populations. World J Microbiol Biotechnol. (2013) 29:301–7. 10.1007/s11274-012-1183-2 23054703

[41] Bekele AZ Koike S Kobayashi Y. Phylogenetic diversity and dietary association of rumen Treponema revealed using group-specific 16S rRNA gene-based analysis. FEMS Microbiol Lett. (2011) 316:51–60. 10.1111/j.1574-6968.2010.02191.x 21204927

[42] Abdul Rahman N. Parks D. H. Vanwonterghem I. Morrison M. Tyson G. W. Hugenholtz P. (2016). A phylogenomic analysis of the bacterial phylum fibrobacteres. Front Microbiol. 6:1469. 10.3389/fmicb.2015.01469 26779135 PMC4704652

[43] Ransom-Jones E Jones DL McCarthy AJ McDonald JE. The fibrobacteres: an important phylum of cellulose-degrading bacteria. Microb Ecol. (2012) 63:267–81. 10.1007/s00248-011-9998-1 22213055

[44] Xue MY Sun HZ Wu XH Guan LL Liu JX. Assessment of rumen bacteria in dairy cows with varied milk protein yield. J Dairy Sci. (2019) 5031–41. 10.3168/jds.2018-15974 30981485

[45] Wallace RJ Rooke JA McKain N Duthie C-A Hyslop JJ Ross DW. The rumen microbial metagenome associated with high methane production in cattle. BMC Genomics. (2015) 16:839. 10.1186/s12864-015-2032-0 26494241 PMC4619255

[46] Cunha CS Veloso CM Marcondes MI Mantovani HC Tomich TR Pereira LGR. Assessing the impact of rumen microbial communities on methane emissions and production traits in Holstein cows in a tropical climate. Syst Appl Microbiol. (2017) 40:492–9. 10.1016/j.syapm.2017.07.008 29113689

[47] Hook S. E. Wright A.-D. G. McBride B. W. (2010). Methanogens: methane producers of the rumen and mitigation strategies. Archaea. 2010:945785. 10.1155/2010/945785 21253540 PMC3021854

[48] Shin EC Choi BR Lim WJ Hong SY An CL Cho KM. Phylogenetic analysis of archaea in three fractions of cow rumen based on the 16S rDNA sequence. Anaerobe. (2004) 10:313–9. 10.1016/j.anaerobe.2004.08.002 16701533

