# Supplementary material for: Bacteroidetes and Firmicutes Drive Differing Microbial Diversity and Community Composition Among Micro-Environments in the Bovine Rumen
Source: Front Vet Sci. 2022 May 19;9:897996. doi: 10.3389/fvets.2022.897996 (PMC9161295; doi:10.3389/fvets.2022.897996)
Supplement: Supplementary file 1 [file Table_1.DOCX]

**Table S1.** Proportion of 16S rRNA gene sequence amplicon sequence variants (ASVs) that were classified at each taxonomic rank.

|  |  | **Taxonomic rank** | | | | |
| --- | --- | --- | --- | --- | --- | --- |
|  | Kingdom | Phylum | Class | Order | Family | Genus |
| ASVs classified | 100.0% | 99.9% | 99.7% | 99.5% | 79.3% | 40.4% |
