# Supplementary material for: Bacteroidetes and Firmicutes Drive Differing Microbial Diversity and Community Composition Among Micro-Environments in the Bovine Rumen
Source: Front Vet Sci. 2022 May 19;9:897996. doi: 10.3389/fvets.2022.897996 (PMC9161295; doi:10.3389/fvets.2022.897996)
Supplement: Supplementary file 2 [file Table_2.DOCX]

**Table S2**. PERMANOVA and PERMDISP results from comparisons between ruminal fluid, pack, and mucosa microbial communities using weighted, generalized, and unweighted UniFrac values. Significant results are bolded (p < 0.05).

| **Test** | **Df** | **SS** | **Pseudo-F** | **R^2^** | **p-adj.** | **PERMDISP (p-adj.)** |
| --- | --- | --- | --- | --- | --- | --- |
| **weighted UniFrac** |  |  |  |  |  |  |
| fluid vs pack | 1 | 0.0032 | 13.7617 | 0.3848 | **0.0001** | 0.0933 |
| fluid vs mucosa | 1 | 0.0032 | 14.6078 | 0.3990 | **0.0001** | 0.4742 |
| pack vs mucosa | 1 | 0.0014 | 5.5276 | 0.2008 | **0.0001** | 0.4901 |
|  |  |  |  |  |  |  |
| **generalized UniFrac** |  |  |  |  |  |  |
| fluid vs pack | 1 | 0.0978 | 9.6456 | 0.3048 | **0.0001** | 0.9058 |
| fluid vs mucosa | 1 | 0.0842 | 8.2717 | 0.2732 | **0.0001** | 0.8871 |
| pack vs mucosa | 1 | 0.0435 | 4.3036 | 0.1636 | **0.0001** | 0.9687 |
|  |  |  |  |  |  |  |
| **unweighted UniFrac** |  |  |  |  |  |  |
| fluid vs pack | 1 | 0.1532 | 3.3540 | 0.1323 | **0.0014** | 0.5840 |
| fluid vs mucosa | 1 | 0.1680 | 3.5333 | 0.1384 | **0.0006** | 0.1615 |
| pack vs mucosa | 1 | 0.1031 | 2.0787 | 0.0863 | **0.0095** | 0.4965 |

Abbreviations: Df, degrees of freedom; SS, sum of squares; p-adj., adjusted p-value
